# Physiology and clinical utility of the peripheral venous waveform

**DOI:** 10.1177/2048004020970038

**Published:** 2020-10-28

**Authors:** Devin Chang, Philip J Leisy, Jenna H Sobey, Srijaya K Reddy, Colleen Brophy, Bret D Alvis, Kyle Hocking, Monica Polcz

**Affiliations:** 1 Department of Biomedical Engineering, Vanderbilt University, Nashville, TN, USA; 2 Department of Anesthesiology, Division of Critical Care, Vanderbilt University Medical Center, Nashville TN, USA; 3 Department of Anesthesiology, Division of Pediatric Anesthesiology, Monroe Carell Jr. Children’s Hospital at Vanderbilt University Medical Center, Nashville TN, USA; 4 Division of Vascular Surgery, Vanderbilt University Medical Center, Nashville TN, USA; 5 Department of Surgery, Vanderbilt University Medical Center, Nashville, TN, USA

**Keywords:** Peripheral venous pressure, waveform analysis, vascular, hemodynamic monitoring

## Abstract

The peripheral venous system serves as a volume reservoir due to its high compliance and can yield information on intravascular volume status. Peripheral venous waveforms can be captured by direct transduction through a peripheral catheter, non-invasive piezoelectric transduction, or gleaned from other waveforms such as the plethysmograph. Older analysis techniques relied upon pressure waveforms such as peripheral venous pressure and central venous pressure as a means of evaluating fluid responsiveness. Newer peripheral venous waveform analysis techniques exist in both the time and frequency domains, and have been applied to various clinical scenarios including hypovolemia (i.e. hemorrhage, dehydration) and volume overload.

## Introduction

A clear understanding of venous waveforms has historically lagged behind that of the arterial system, primarily due to the venous waveform being low in amplitude and highly susceptible to noise. Recent advancements in amplifying technology have allowed for improvement in venous waveform capture. Analysis of venous waveforms has revealed insight into intravascular volume status with clinical implications toward determination of dehydration, hemorrhage, and volume overload in diseases such as heart failure and renal failure (Table 1).^2–10^

## Methods

We conducted a review of the literature to explore the physiology, acquisition, analysis, and applications of peripheral venous waveforms. PubMed, AccessMedicine, and the USPTO databases were searched for relevant research with a focus on venous waveforms. Search terms used include peripheral venous waveform, peripheral venous pressure (PVP), central venous pressure (CVP), volume status monitoring, plethysmography, venous pressure, hypervolemia, hemorrhage, hypovolemia, and peripheral capillary wedge pressure.

## Physiology of venous waveforms

The venous system can be divided into two major compartments: the central venous conduit containing ∼18% of the total blood volume (TBV), and the reactive venous reservoir containing ∼45% TBV.^11^ The latter compartment’s high compliance (110 mL/mmHg peripheral venous compartment vs 4 mL/mmHg central venous compartment)^12^ results in a high capacitance to store blood. Neurohumoral input controls venous tone, resulting in vasodilation or vasoconstriction to shift blood between the central and reactive compartments in order to maintain stroke volume.

**Figure 1.**
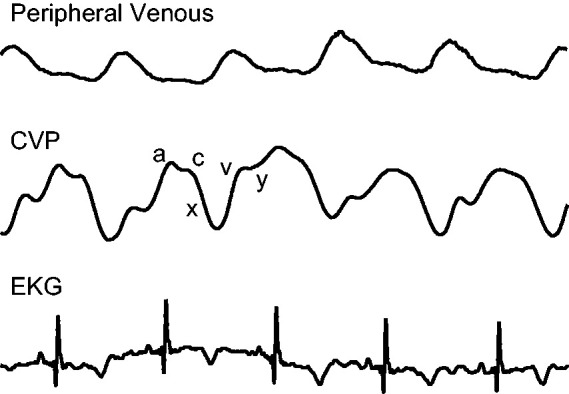
Comparison of peripheral venous and central venous (CVP) waveforms, time-synced with EKG. CVP consists of three peaks (a,c,v) and two descents (x,y).

Mean CVP is one measure frequently used to estimate intravascular volume and guide fluid resuscitation in intensive care unit (ICU) settings. The CVP waveform consists of three peaks and two descents (Figure 1) related to different stages of the cardiac cycle. The various peaks and troughs of the CVP tracing represent forward and backward waves as blood flows through the heart. These waves are easily seen on a CVP tracing, but the backward compression waves are not prominent in the peripheral venous system. Thus, the peripheral venous waveform morphology differs from that of the central venous system (Figure 1). The differences in the peripheral venous waveform may be due to the direction of the waveform (forward vs. backward compression wave), dampening related to the presence of valves, increased compliance, and/or distance from the heart. Additionally, mean pressure is higher in the peripheral venous system (mean PVP 5.5 mmHg vs mean CVP 4.6 mmHg).^13^ Because the peripheral (reactive) venous system serves as the body’s main volume reservoir, analysis focused on this specific compartment has yielded information toward estimation of intravascular volume changes and thus will be the focus of this review.^14,15^

## Methods of venous waveform acquisition

Acquisition of venous waveforms has been accomplished through a variety of techniques. One of the earliest methods of venous waveform capture was via direct transduction using a peripheral intravenous catheter connected to a pressure transducer.^1,2,6,7^ Furthermore, a piezoelectric sensor placed on the volar aspect of the wrist, directly over the superficial veins, has also allowed for non-invasive capture of the peripheral venous waveform.^3,4,16,17^ The piezoelectric sensor is connected to a control box that amplifies the venous waveform detected from vibrations related to the low amplitude pulsatile flow of venous blood. In addition to direct invasive and non-invasive capture of peripheral venous waveforms, indirect analysis of plethysmographic waveforms have been used to detect the peripheral venous waveform. Shelley et al. reported the use of a pulse oximeter to record the plethysmographic waveform on several patients. Comparison of this waveform to both the peripheral venous waveform (intravenous capture) and arterial tracings suggest that diastolic plethysmographic peaks correlate with peripheral venous pulsation.^18^ This was confirmed with loss of diastolic peaks with occlusion of venous return.

## Techniques for waveform analysis

Different methods of analysis have been applied toward assessment of intravascular volume based on the peripheral venous waveform. Respiratory variation is one method of waveform analysis used to predict fluid responsiveness focusing on the plethysmographic waveform. The Plethysmography Variability Index (PVI, Masimo Corporation, Irvine, California, USA) is a method designed to automatically calculate respiratory variations in plethysmographic waveforms.^19^ PVI calculates the rate of change in perfusion index during complete respiratory cycles. The perfusion index is calculated from the differences between the absorption of light by the local static tissues and the variable absorption of light by the arterial inflow. PVI has been shown to predict fluid responsiveness in mechanically ventilated patients, limiting its practicality.^19^ Alian et al. analyzed multiple parameters related to directly transduced peripheral venous waveforms obtained under progressive lower body negative pressure (LBNP), simulating a hypovolemic state. These include the time-domain parameters of venous pulse pressure, mean venous pressure, pulse width, maximum and minimum slope over the whole peak, as well as frequency domain analysis of the cardiac and respiratory modulations. Progressive LBNP resulted in a reduction of the cardiac but not respiratory amplitude density, as well as a significant reduction in all other time-domain measures prior to detectable hemodynamic changes such as blood pressure.^20^

**Figure 2.**
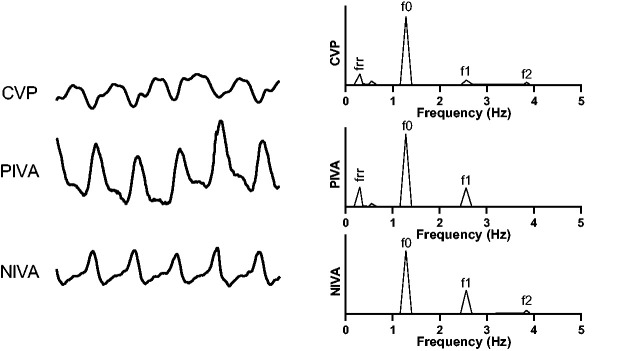
Raw venous waveforms in the time-domain (left) and frequency domain (right). The top is a central venous waveform, followed by a peripheral venous waveform acquired from direct transduction (middle) and noninvasively with a piezoelectric sensor (bottom).f0 corresponds to the fundamental frequency which is equal to the pulse rate. Higher harmonics of the pulse rate are denoted by f1 and f2, as well as a low frequency component (frr) corresponding to the respiratory rate.

A number of other studies have utilized the amplitude changes of the cardiac frequencies to refine frequency-domain analysis of peripheral venous waveforms for volume assessment. Similar to other vascular waveforms, the raw venous signal is characterized most prominently by a wave generated by the cardiac cycle. Although this fundamental frequency (equal to the pulse rate) is easily observed by eye in the time-domain, deconvolution of the waveform into the frequency domain with a Fourier transformation exposes additional frequencies which are not otherwise visible – this includes a wave (low-frequency) generated by the respiratory cycle, as well as higher harmonics, or multiples, of the pulse rate (Figure 2). The higher harmonics indicate that the waveform is more complex than a pure sin/cosine composition. The underlying peripheral venous waveform is hypothesized to contain elements of the CVP waveform^21^ and local resonance through the tissues surrounding the vein have also been hypothesized to propagate these higher harmonics. Ratiometric algorithms incorporating the relative amplitude or power contributions of these cardiac frequencies to their overall sum have been developed and validated in states of hypo- and hypervolemia. ^1–4,6,7,16,17^ These algorithms are referred to as Peripheral IntraVenous waveform Analysis (PIVA) in directly transduced waveforms^1,2,6,7^ and NonInvasive Venous waveform Analysis (NIVA) in waveforms obtained non-invasively with a piezoelectric sensor placed on the wrist.^3,4,16,17^ A number of other algorithms in the frequency domain have been developed and tested, including calculation of overall signal power or tracking changes in the amplitude of the fundamental pulse frequency across intravascular volume events.^1,2,5,6^

**Table 1.** Summary of key studies reviewed in this article.

Study	PVP capture method	Application/demographic	Comparators	Primary finding
Hocking et al.^1^	PIVA (IV Catheter, frequency domain)	Acute hemorrhage detection, porcine (n = 8)	HR, MAP, SI	F1 amplitude correlated with volume of hemorrhage, euvolemia return, and iatrogenic fluid overload. HR and MAP did not.
Hocking et al.^2^	PIVA (IV Catheter, frequency domain)	Adults undergoing hemodialysis (n = 37)	PVP, BP, HR	Change in PIVA signal correlated linearly with volume removed during dialysis (R^2^=0.77). and predicted
Alvis et al.^3^	NIVA (piezo, frequency domain)	Acute hemorrhage detection, adults (n = 50), porcine (n = 7)	HR, CVP, CO, PAD, PCWP	NIVA predicts 500 mL blood loss in adults (92% sensitivity, 84% specificity).In porcine model, PAD, PCWP, NIVA changed with 200 mL loss (P < 0.05).
Alvis et al.^4^	NIVA (piezo, frequency domain)	Estimation of intracardiac filling pressure in adults undergoing RHC (n = 96)	PCWP	NIVA demonstrated linear correlation with PCWP (r = 0.69, P < 0.05) ranging from 4-40mmHg
Bonasso et al.^5^	IV Catheter (frequency domain)	Assessment of dehydration, pediatric (n = 18)	n/a	Frequency domain analysis of PVP predicted dehydration with 97.75% sensitivity and 93.07% specificity
Bonasso et al.^6^	IV Catheter (frequency domain)	Acute hemorrhage detection, porcine (n = 9)	HR, SBP	10% blood loss led to significant changes in the amplitude of F1 (18.87%, p < 0.01), but not SBP or HR.
Miles et al.^7^	PIVA (IV Catheter, frequency domain)	Assessment of diuresis in adults with ADHF (n = 14)	Brain natriuretic peptide, chest radiographic measures	PIVA correlates with volume removed during diuresis (R^2^=0.781) and predicts readmissons.
Sobey et al.^8^	NIVA (piezo, frequency domain)	*Intraoperative volume status monitoring in pediatric complex cranial vault reconstruction patients (n = 14)*	MAP	NIVA correlates with net change in volume (r=0.67, p < 0.05) while MAP does not
Polcz et al.^9^	NIVA (piezo, frequency domain)	*Assessment of vasoactive agents effecting PVP waveforms, porcine (n = 8)*	PCWP, MAP	*Phenylephrine (vasoconstrictor) causes a significant decrease in NIVA, increase in MAP, and no change in PCWP. Sodium nitroprusside (vasodilator) causes a significant increase in NIVA, decrease in MAP, and no change in PC*
Alvis et al.^10^	NIVA (piezo, frequency domain)	*Volume status assessment in adults undergoing hemodialysis (n = 38)*	n/a	*Significant decreases in NIVA after dialysis (1.203 vs 0.868, p < 0.05) and predicted intradialytic hypotension with 80% sensitivity and 100% specificity (n = 16, AUC0.87, p < 0.05).*
Shelley et al.^19^	PPG	Intraoperative monitoring in adults undergoing various operations (n = 3)	BP	PPG diastolic peak disappeared and significant blood pressure variability occurred with blood loss.Strong association between peripheral venous pulse and diastolic peaks in PPG observed.
Alian et al.^21^	IV Catheter (time domain and frequency domain)	Assessment of hypovolemia simulated with LBNP (n = 11)	HR, BP	Significant reduction in PVP waveform at ∼30mmHg LBNP, HR increased significantly, no change in BP.

## Clinical applications of venous waveforms

One of the largest clinical applications of volume status assessment is the detection of hemorrhage, which is traditionally difficult to identify clinically. Changes in vital signs are delayed due to neurohumoral compensatory mechanisms that maintain cardiac output until significant blood loss has occurred (∼25–35% blood loss).^22,23^ Analysis of the arterial waveform has been proposed to detect hypovolemia but requires mechanical ventilation with high tidal volumes for accuracy, limiting its practicality for use in hemorrhage states and for triage.^24^ Peripheral venous waveforms, on the other hand, demonstrate more promise. In a case report of three patients, Shelley et al. demonstrated loss of the diastolic peak, which correlates with the peripheral venous waveform, in the plethysmographic signal with progressive blood loss during surgery.^18^ As discussed earlier, Alian et al. demonstrated reduction in venous pulse pressure, mean venous pressure, pulse width, maximum and minimum slope, and amplitude density of the cardiac frequency with LBNP, which simulates a hypovolemic state, prior to changes in vital signs.^20^ Bonasso et al. demonstrated that acute hemorrhage of 10% blood volume in a porcine model resulted in a significant decrease in the fundamental pulse frequency amplitude of the venous waveform while heart rate and systolic blood pressure did not significantly change.^6^ Hocking et al. demonstrated that PIVA was able to detect minor blood loss (200 mL, ∼7% TBV) in a porcine hemorrhage model, while blood pressure and heart rate did not significantly change up to 400 mL blood loss (∼12% TBV).^1^ Alvis et al. reported that NIVA could accurately detect 500 mL (∼8%) blood loss in healthy blood donors with a sensitivity of 92% and specificity of 84%, and changes in NIVA correlated with blood loss in a porcine model prior to observed changes in vital signs.^3^ Dehydration, another clinical syndrome characterized by hypovolemia, has also been accurately assessed using transduced peripheral venous waveforms in pediatric patients. Overall signal power in the frequency domain was reduced in hypovolemic patients compared to resuscitated patients, with an algorithm developed to predict dehydration that demonstrated 100% sensitivity and specificity.^5^ In pediatric patients undergoing complex cranial vault reconstruction, Sobey et al. found a linear correlation between NIVA and a net change in volume calculated as a sum of blood loss and fluid/blood administration normalized to body weight (r = 0.67, p < 0.05, n = 14). In the same study MAP, a widely used indicator of volume status in anesthetized children, did not correlate with volume change (r=-0.09, p > 0.05, n = 14).

In addition to applications toward hypovolemia, such as hemorrhage and dehydration, venous waveforms also have utility in detection of volume overload, otherwise known as hypervolemia. Examples of clinical syndromes that lead to pathologic volume overload and congestion include congestive heart failure, end stage renal disease, or overly aggressive fluid resuscitation in perioperative and critically ill patients. This results in tissue and pulmonary edema in addition to decreased cardiac output due to overstretching of the myocardium. Pulmonary capillary wedge pressure (PCWP) obtained with a Swan-Ganz catheter represents the current gold standard in clinical volume status assessment, reflecting left sided cardiac filling pressures, a surrogate for volume status. This requires central venous catheterization, limiting its usage to the hospital setting and carrying potential procedural risks.

CVP, among other hemodynamic measurements, has been used to guide diuresis in volume-overloaded patients. Sperry et al. investigated the usage of PVP as a less invasive alternative to CVP and found a strong correlation (r = 0.95, n = 30), consistent with previous studies (0.88≤ r ≤ 0.93). ^25,26^ However, PVP was only moderately correlated with PCWP (r = 0.56), suggesting an underlying weakness in using mean venous pressures to predict intravascular volume.^25^ CVP as an indicator of fluid responsiveness in hypovolemic patients is also suspect. A meta-analysis performed by Marik et al. combined patient data from multiple studies evaluating the sensitivity and specificity of CVP against cardiac preload, defining fluid responsiveness as an increase in stroke volume index (SVI) or cardiac index (CI) by 15% following a 500 mL fluid challenge.^27^ 57%±13% of all patients were fluid responders (n = 1802), with an area under the curve (AUC) for CVP to predict fluid-responsiveness of 0.56. Similar AUC values resulted when the data was separated into ICU, operating room, cardiac, or noncardiac patients. AUC values resulted when the data was separated into ICU, operating room, cardiac, or noncardiac patients.

More sophisticated peripheral venous analysis has potential to serve as a less invasive alternative to PCWP for assessment of volume overload. In a porcine volume overload model, PIVA could detect small increases in volume (200 mL), and was more sensitive than heart rate or blood pressure.^1^ In patients undergoing hemodialysis, a strong correlation between percent change in PIVA values and ultrafiltration volumes was reported (R^2^=0.77, n = 31), with no significant correlations between volume removed and PVP, blood pressure, or heart rate.^2^ Comparison of the least-squares slopes of NIVA on hemodialysis patients prior to onset of intradialytic hypotension (IDH) to slopes on patients without IDH showed a 80% sensitivity and 100% specificity in predicting IDH (AUC 0.87, p < 0.05, n = 16). Furthermore, there was a significant decrease in NIVA value before and after dialysis (1.203 vs 0.868, p < 0.05, n = 38).^10^ In patients admitted with acute decompensated heart failure, a significant correlation between volume removed during diuresis and change in PIVA signal (R^2^=0.78, n = 14) was also demonstrated.^7^ NIVA, which has the benefit of using a completely non-invasive sensor for waveform acquisition, has demonstrated strong correlation with PCWP in patients undergoing right heart catheterization (r = 0.69, p < 0.05, n = 83) and is able to predict PCWP > 18 mmHg, a clinically accepted value indicating venous congestion,^28,29^ with a sensitivity of 80% and a specificity of 53%.^4^

## Limitations of venous waveform analysis

The low amplitude nature of the venous signal represents the main limitation to translation into clinical practice. Signal noise may be introduced by surgical electrocautery and patient or transducer movement.^3,4,30,31^ Ideally, waveform recordings with motion artifact have the noise either automatically or manually removed.^14,30,31^ This is usually not feasible, resulting in the large number of signals excluded from many of these studies. Cardiac arrhythmias, such as atrial fibrillation, common in patients with volume overload, may also preclude analyses in the frequency domain due to irregularities in the frequency peaks.^4^ In addition to intravascular volume, other physiological parameters may affect the venous waveform and thus its analysis, such as venous tone. Polcz et al. investigated the effect of vasoactive agents on waveform analysis and calculation of a NIVA value, finding that phenylephrine, a vasoconstrictor, increased the power of f_1_ relative to f_0_ causing an underestimation in volume status. Sodium nitroprusside, a vasodilator, had the opposite effect and decreased the power of f_1_ relative to f_0_ and caused an overestimation of volume.^9^ Further studies are needed to better understand the effects vasoactive and vasodilatory agents have on these volume assessment techniques.

## Conclusion

Many recent advancements have been made in the field of invasive and noninvasive capture of peripheral venous waveforms. A number of methods for analysis of these waveforms have shown promise toward assessing intravascular volume in a variety of clinical conditions. Advancements to limit noise and motion artifact and additional studies demonstrating improved clinical outcomes are needed to support widespread clinical use of venous waveform analysis for determination of volume status in adult and pediatric patients.

## Physiology and Clinical Utility of the Peripheral Venous Waveform

**Table:** 

Abbreviation	Definition
ADHF	Acute Decompensated Heart Failure
AUC	Area Under Curve
BDW	Backwards Decompression Wave
BP	Blood Pressure
CI	Cardiac Index
CVP	Central Venous Pressure
EKG	Electrocardiogram
fFT	Fast Fourier Transform
HR	Heart Rate
ICU	Intensive Care Unit
IDH	IntraDialytic Hypotension
IV	IntraVenous
LBNP	Lower Body Negative Pressure
MAP	Mean Arterial Pressure
NIVA	Non-Invasive Venous waveform Analysis
PAD	Pulmonary Artery Diastolic pressure
PCWP	Pulmonary Capillary Wedge Pressure
PIVA	Peripheral IntraVenous waveform Analysis
PPG	PhotoPlethysmoGraph
PVI	Pleth Variability Index
PVP	Peripheral Venous Pressure
SBP	Systolic Blood Pressure
SI	Shock Index
SVI	Stroke Volume Index
TBV	Total Blood Volume
